# The Association Between the HALP Score and Glycemic Control in Older Adults with Type 2 Diabetes Mellitus Receiving Home Health Care

**DOI:** 10.3390/healthcare14152272

**Published:** 2026-07-25

**Authors:** Safiye Kübra Çetindağ Karatlı, Münevver Yıldız, Ebru Uğraş, Erhan Şimşek, Ahmet Keskin

**Affiliations:** 1 Department of Family Medicine, Ankara Yıldırım Beyazıt University, Ankara 06220, Türkiye; 2 Department of Family Medicine, Ankara Bilkent City Hospital, Ankara 06800, Türkiye

**Keywords:** type 2 diabetes mellitus, HALP score, glycemic control, home health care, inflammation

## Abstract

Background: The hemoglobin, albumin, lymphocyte, and platelet (HALP) score is a simple inflammation–nutrition index that has been associated with adverse outcomes in patients with type 2 diabetes mellitus (T2DM). This study investigated the association between the HALP score and poor glycemic control in older adults with T2DM receiving home health care after excluding patients with malnutrition. Methods: This retrospective, cross-sectional study included 201 patients aged ≥65 years with T2DM whose routinely collected home health care records between 15 December 2025 and 15 March 2026 were retrospectively reviewed after ethics committee approval. Poor glycemic control was defined as HbA1c ≥ 8.0%. Logistic regression analyses were performed to identify factors associated with elevated HbA1c levels. A sensitivity analysis including clinically relevant variables was additionally conducted. The discriminative performance of HALP was evaluated using receiver operating characteristic (ROC) curve analysis. Results: The median age was 83 years, 52.2% were male, and 51 patients (25.4%) had elevated HbA1c levels (≥8.0%). In univariate analysis, high HALP was significantly associated with lower odds of elevated HbA1c levels (OR: 0.27; 95% CI: 0.14–0.52; *p* < 0.001), whereas dementia showed a borderline association (OR: 0.36; 95% CI: 0.12–1.06; *p* = 0.065). In multivariable analysis, high HALP remained independently associated with lower odds of poor glycemic control (OR: 0.28; 95% CI: 0.14–0.54; *p* < 0.001). In a sensitivity analysis adjusted for age, sex, Barthel group, and dementia, high HALP maintained its independent association with lower odds of elevated HbA1c levels (OR: 0.30; 95% CI: 0.15–0.59; *p* = 0.001). The HALP score demonstrated weak to modest discriminative ability for predicting HbA1c ≥ 8.0% (AUC: 0.645; 95% CI: 0.552–0.739; *p* = 0.002), with 75.3% sensitivity and 45.1% specificity at a cut-off value of 29.24. Conclusions: High HALP was independently associated with better glycemic control in older adults with T2DM receiving home health care. However, given its weak to modest discriminative ability, HALP should be interpreted alongside clinical assessment and routine metabolic parameters rather than as a standalone diagnostic or predictive criterion.

## 1. Introduction

Type 2 diabetes mellitus (T2DM) is a chronic metabolic disease with rapidly increasing global prevalence. According to the International Diabetes Federation (IDF) Diabetes Atlas, 11th edition, the global age-standardized prevalence among adults (20–79 years) reached 11.1% in 2025, while in Türkiye, it was reported as 16.5% [1,2]. Diabetes management is particularly challenging in older adults due to multimorbidity, sarcopenia, functional dependence, and chronic low-grade inflammation (inflammaging).

Current guidelines recommend individualizing glycemic targets in older adults. The American Diabetes Association (ADA) 2026 Standards of Care suggest more flexible HbA1c targets (generally <8.0–8.5%) for older patients with multiple comorbidities and limited life expectancy [3].

In recent years, composite biomarkers reflecting both inflammatory and nutritional status have gained importance in geriatric diabetes care. The hemoglobin, albumin, lymphocyte, and platelet (HALP) score is a simple, routinely available index that integrates these components. The HALP score provides a broad assessment of systemic inflammation, nutritional status, and immune response [4,5]. Systemic inflammation is closely involved in insulin resistance and metabolic dysregulation, whereas nutritional status may influence glucose metabolism, frailty, and the ability to maintain stable glycemic control in older adults. Therefore, HALP may be relevant to glycemic control by reflecting both inflammatory activity and nutritional reserve. Previous studies have linked lower HALP scores with diabetic complications (retinopathy, nephropathy), frailty, and increased mortality in patients with T2DM [6,7].

However, the majority of existing research has been conducted in hospital or community-dwelling populations. These settings may not fully reflect the unique clinical profiles and bio-nutritional challenges of individuals who are homebound. Older adults receiving home health care represent a distinct and vulnerable population characterized by advanced age, functional dependence, and a high burden of multimorbidity [8]. Data on the clinical utility of the HALP score in older adults with T2DM receiving home health care—particularly in a nutritionally adequate (MNA ≥ 24) but functionally dependent group—remain limited [9]. Therefore, this retrospective cross-sectional study aimed to investigate the association between the HALP score and poor glycemic control (HbA1c ≥ 8.0%) in patients aged 65 years and older with T2DM receiving home health care services. We hypothesized that lower HALP scores would be independently associated with poor glycemic control in this population.

## 2. Materials and Methods

### 2.1. Study Design and Participants

This retrospective cross-sectional study was conducted using anonymized routinely collected clinical data from the Home Health Care Unit of Ankara Bilkent City Hospital. The study population was identified from patients aged ≥65 years with T2DM who had received home health care services between 15 December 2025 and 15 March 2026. This three-month period was selected to ensure temporal consistency between home health care assessments and laboratory measurements and to minimize heterogeneity related to changes in clinical practice or follow-up conditions. Because HbA1c reflects average glycemia over approximately the preceding 8–12 weeks, the selected period was considered clinically relevant for evaluating glycemic control. Data extraction and analysis for research purposes were performed only after ethics committee approval was obtained on 25 March 2026. The study was reported in accordance with the Strengthening the Reporting of Observational Studies in Epidemiology (STROBE) guidelines for cross-sectional studies.

The study protocol was approved by the Ankara Bilkent City Hospital Clinical Research Ethics Committee (Decision No.: TABED 1-26-2372; Date: 25 March 2026) and conducted in accordance with the principles of the Declaration of Helsinki. Due to the retrospective design and use of anonymized data, the requirement for informed consent was waived.

Inclusion criteria were as follows:

- Age ≥ 65 years;
- Confirmed diagnosis of T2DM;
- Received home health care services at least once during the clinical care period;
- Availability of HbA1c, complete blood count, and serum albumin levels from the first visit;
- Mini Nutritional Assessment (MNA) score ≥ 24 (normal nutritional status).

Nutritional status was evaluated using the Mini Nutritional Assessment (MNA), a validated screening tool for older adults. The total MNA score ranges from 0 to 30, and a score of ≥24 indicates normal nutritional status; therefore, only patients with MNA ≥ 24 were included to minimize potential confounding related to malnutrition [9,10].

**Figure 1:**
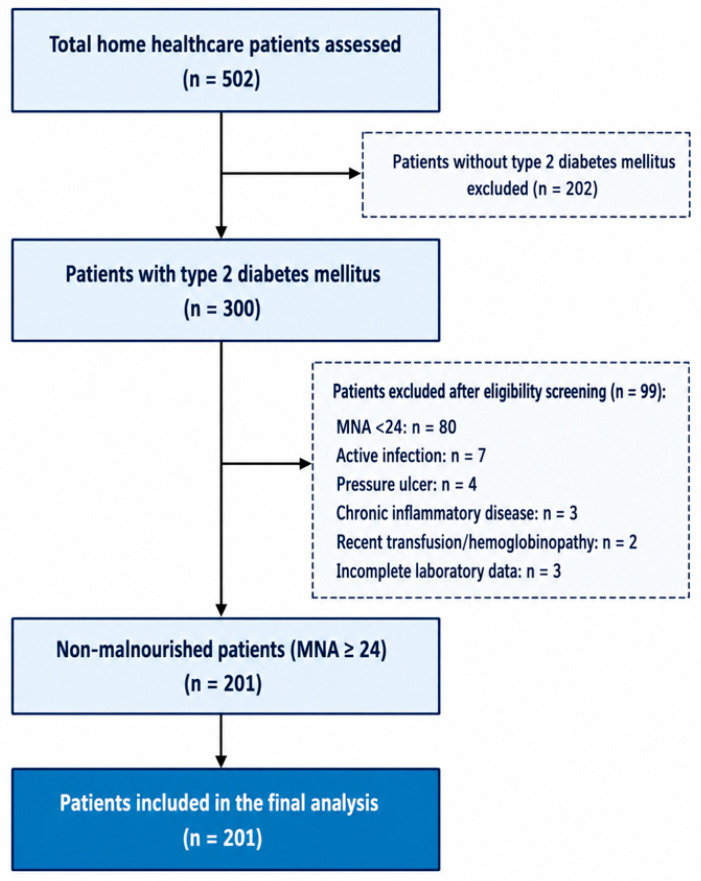
Flow diagram of the patient selection process.

Of the 502 patients evaluated under the home health care program, 300 had T2DM. After applying the inclusion criteria, 201 patients were included in the final analysis. The patient selection process is shown in Figure 1.

Exclusion criteria were as follows:

- Autoimmune or chronic inflammatory diseases (e.g., rheumatoid arthritis, vasculitis);
- Active infection;
- Pressure ulcers;
- Blood transfusion within the last 3 months or hemoglobinopathy;
- Incomplete laboratory data for HALP calculation.

### 2.2. Clinical and Laboratory Assessments

Demographic data (age, sex), comorbidities (hypertension, coronary artery disease, hyperlipidemia, asthma/COPD [chronic obstructive pulmonary disease], dementia), and functional status were retrieved from the hospital information system. Functional status was assessed using the most recent Barthel Index score recorded during home health care follow-up [11].

All laboratory parameters used in the analyses, including complete blood count, serum albumin, and HbA1c, were obtained from blood samples collected on the exact same day as the initial eligible home health care assessment. To ensure absolute clinical and temporal consistency, the Mini Nutritional Assessment (MNA) was also performed and recorded on this same date. Consequently, all clinical assessments and laboratory measurements were fully concurrent, reflecting a single-day time window for each patient’s baseline data. For patients with multiple visits, only the first assessment with this complete, concurrent same-day dataset was included.

Hemoglobin values were converted from g/dL to g/L (multiplied by 10) for HALP calculation. The HALP score was computed using the formula: HALP score = (hemoglobin [g/L] × albumin [g/L] × lymphocytes [×10^9^/L])/platelets [×10^9^/L] [4,5].

Complete blood count parameters, including hemoglobin, lymphocyte count, and platelet count, were measured using an automated hematology analyzer in the Central and Emergency Laboratories of Ankara Bilkent City Hospital. Serum albumin levels were measured using standard automated biochemical methods. HbA1c was measured using high-performance liquid chromatography (HPLC) with methods aligned with the National Glycohemoglobin Standardization Program (NGSP) and International Federation of Clinical Chemistry and Laboratory Medicine (IFCC) standards. All laboratory results were extracted from the hospital information system. For patients with multiple available measurements during the study period, only the first complete set of laboratory values obtained at the first home health care assessment was used to ensure consistency. Patients with missing HbA1c, complete blood count, serum albumin, or MNA data were excluded from the analysis; therefore, no imputation was performed.

### 2.3. Outcome Definition

Poor glycemic control was defined as HbA1c ≥ 8.0%, in accordance with the American Diabetes Association guidelines recommending more flexible targets in older adults with comorbidities.

### 2.4. Statistical Analysis

The performance of the HALP score in predicting elevated HbA1c levels was evaluated using Receiver Operating Characteristic (ROC) curve analysis, and the optimal cut-off value was determined as the point where the Youden index was maximized. Based on the cut-off value of 29.24 obtained from this analysis, the HALP score was categorized, and patients were divided into “low HALP” and “high HALP” groups for use in logistic regression analyses. This dichotomization was performed to improve clinical interpretability. However, because dichotomization of continuous variables may reduce statistical power and lead to loss of information, the ROC-derived cut-off should be considered exploratory rather than definitive, and the findings were interpreted cautiously [12,13].

Since continuous variables did not follow a normal distribution in statistical analyses, they are expressed as the median (minimum–maximum), while categorical variables are expressed as counts and percentages (%). The Mann–Whitney U test and the chi-square test were used for comparisons between groups. Univariate and multivariate logistic regression analyses were performed to identify factors associated with elevated HbA1c levels. Variables found to be significant at the *p* < 0.10 level in the univariate analysis were included in the multivariate model. Results are presented as odds ratios (ORs) and 95% confidence intervals. Statistical significance was set at *p* < 0.05. All analyses were performed using IBM SPSS Statistics (version 25.0). Model calibration was assessed using the Hosmer–Lemeshow goodness-of-fit test. Multicollinearity was evaluated using variance inflation factor (VIF) values. No missing data were present for the variables included in the analysis.

Age was included in the logistic regression analyses as a continuous variable to avoid information loss and to provide an estimate per one-year increase.

Due to the retrospective cross-sectional design of the study, the sample size was not determined a priori. All eligible patients who met the inclusion criteria and had complete data during the predefined study period were included in the study. However, a post hoc power analysis was performed using G*Power software version 3.1.9.7 to evaluate the statistical adequacy of the available sample size of 201 patients. Based on the observed association between HALP groups and poor glycemic control, with a type I error rate (alpha) of 0.05, a baseline prevalence of poor glycemic control of 35% in the reference group, and the observed effect size parameters, the total sample size of 201 patients achieved an estimated statistical power (1-β) of approximately 84.2% to detect the reported differences. Given the inherent limitations of post hoc power analyses in retrospective designs, these findings should be interpreted as supportive and exploratory and warrant validation in larger prospective studies.

## 3. Results

A total of 201 patients with T2DM were included in the study. The median age of the patients was 83 (65–100) years, and 52.2% were male. The vast majority of patients had hypertension (93.5%) and at least one comorbidity (97.5%). Overall, 51 patients (25.4%) had elevated HbA1c levels (≥8.0%), whereas 150 patients (74.6%) had HbA1c levels below 8.0%. According to the Barthel Index, most patients were classified as severely (61.7%) or moderately (21.9%) dependent (Table 1).

Upon examination of the laboratory parameters, the median hemoglobin level was 12.6 g/dL, the median albumin level was 39 g/L, and the median lymphocyte count was 1.89 × 10^9^/L. The median HbA1c level was 6.8% (4.7–12.0). The median HALP score was 37.4 (3.4–145.1) (Table 2).

In univariate logistic regression analysis, the prevalence of elevated HbA1c (≥8.0%) was found to be statistically significantly higher in patients with low HALP scores compared to those with high HALP scores (OR: 0.27; 95% CI: 0.14–0.52; *p* < 0.001). No significant association was observed between HbA1c levels and other variables, including age, sex, Barthel Index, coronary artery disease, and asthma/COPD. A borderline significant association was found between the presence of dementia and low HbA1c levels (OR: 0.36; 95% CI: 0.12–1.06; *p* = 0.065) (Table 3). Hypertension was not included in the multivariable analyses because it was present in 93.5% of the cohort, resulting in limited variability and imprecise estimation of its independent association. Therefore, it was excluded for statistical reasons rather than because of a lack of clinical relevance [14].

Variables that were significant at the *p* < 0.10 level in the univariate analysis (HALP score and dementia) were included in the multivariate model. In the multivariate analysis, only the HALP score remained independently associated with poor glycemic control after adjustment for available covariates (OR: 0.28; 95% CI: 0.14–0.54; *p* < 0.001). The presence of dementia lost its independent significance (*p* = 0.095) (Table 3).

In a sensitivity analysis including clinically relevant variables regardless of their univariate statistical significance, high HALP remained independently associated with lower odds of elevated HbA1c levels after adjustment for age, sex, Barthel group, and dementia (OR: 0.30; 95% CI: 0.15–0.59; *p* = 0.001) (Table 4).

The logistic regression models demonstrated adequate calibration according to the Hosmer–Lemeshow goodness-of-fit test, and no significant multicollinearity was detected based on VIF values.

**Figure 2:**
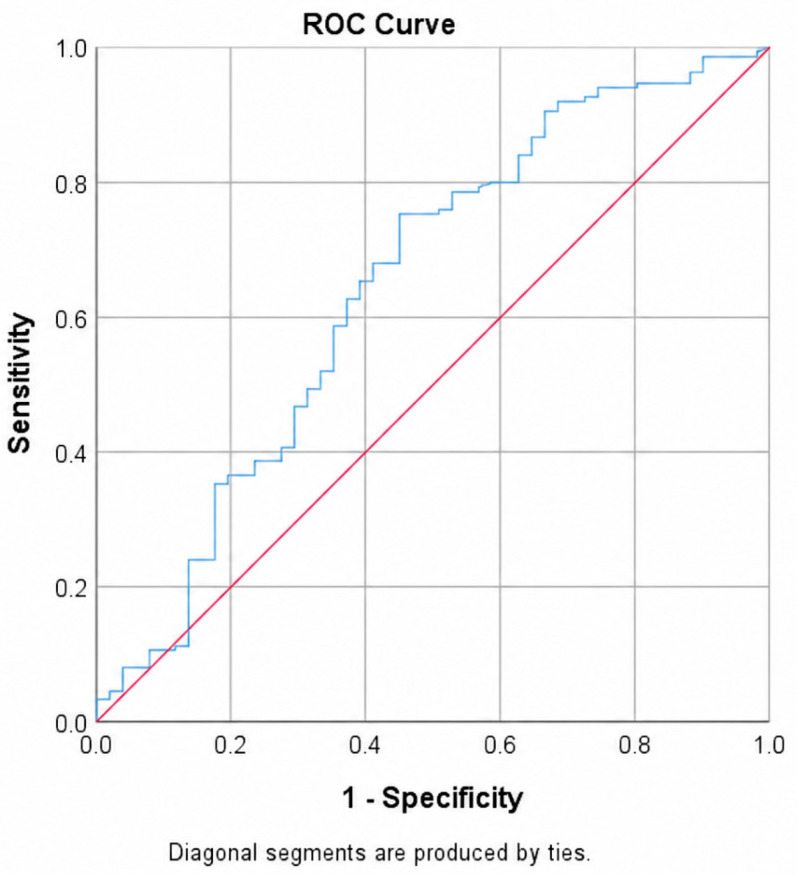
Receiver operating characteristic (ROC) curve of the HALP score for predicting elevated HbA1c levels. The area under the curve (AUC) was 0.645 (95% CI: 0.552–0.739; *p* = 0.002).

The predictive performance of the HALP score for HbA1c levels was evaluated using ROC analysis. The area under the curve (AUC) value for the HALP score in predicting elevated HbA1c levels was found to be 0.645 (95% CI: 0.552–0.739; *p* = 0.002), indicating weak to modest discriminative performance. The optimal cut-off value for the HALP score was 29.24, yielding a sensitivity of 75.3% and a specificity of 45.1% (Figure 2).

## 4. Discussion

In the present study, a low HALP score was independently associated with elevated HbA1c levels in older adults with T2DM receiving home health care without malnutrition, indicating an increased likelihood of poor glycemic control. The HALP score demonstrated weak to modest discriminative ability for elevated HbA1c levels. Therefore, given its limited discriminative performance, HALP should be interpreted alongside clinical assessment and routine metabolic parameters rather than as a standalone diagnostic or predictive criterion. To our knowledge, this is among the few studies evaluating this relationship in older adults receiving home health care. This association may reflect the interplay between chronic inflammation, impaired immune response, and metabolic dysfunction, all of which are closely linked to insulin resistance. Chronic low-grade inflammation and oxidative stress play a central role in the pathogenesis of T2DM, and their interaction with hyperglycemia further worsens metabolic control [15,16].

Previous studies have shown significant associations between various inflammatory indices, including the neutrophil-to-lymphocyte ratio (NLR), platelet-to-lymphocyte ratio (PLR), and systemic immune–inflammation index (SII), and poor glycemic control, insulin resistance, elevated HbA1c, diabetic complications, and mortality [17,18,19,20,21,22,23]. However, unlike NLR and PLR, which are derived from blood cell counts, the HALP score incorporates hemoglobin and albumin in addition to lymphocyte and platelet counts. Because anemia, nutritional vulnerability, and inflammation are common clinical issues in older adults receiving home health care, these additional components may be relevant when evaluating inflammation–nutrition status in this population [24,25]. Consistent with these findings, our study found that a low HALP score was significantly associated with poor glycemic control (HbA1c ≥ 8.0%).

The HALP score reflects systemic inflammation, immune status, and nutritional condition. It has been linked to adverse outcomes such as poor survival, prolonged hospitalization, low muscle mass, increased mortality, and cardiovascular complications in patients with diabetes [4,24,25,26,27]. Our findings suggest that HALP may also be associated with metabolic control.

Although the HALP score was independently associated with elevated HbA1c, its weak to modest discriminative ability (AUC 0.645) indicates limited standalone predictive performance. Accordingly, HALP should be interpreted alongside clinical assessment and routine metabolic parameters rather than as an independent diagnostic or predictive criterion. Furthermore, the association between HALP and glycemic control remained significant in the sensitivity analysis adjusted for age, sex, functional dependency, and dementia, supporting the consistency of the observed association.

Functional dependence is an important clinical factor that may influence glycemic control in older adults with T2DM. Previous studies have suggested that dependence in activities of daily living may complicate diabetes management through reduced physical activity, irregular nutrition, decreased medication adherence, and a greater need for caregiver support [28]. However, in the present study, the Barthel Index was not significantly associated with poor glycemic control. This finding may partly reflect the limited variability in functional status, as most patients in our cohort were already moderately or severely dependent. In addition, HbA1c levels may have been influenced more strongly by factors such as antidiabetic treatment, medication adherence, dietary patterns, and caregiver support than by functional dependence itself.

This study evaluated a predominantly geriatric and functionally dependent home-care population. A major strength of this study is its focus on older adults with T2DM receiving home health care—a vulnerable population with high levels of inflammation and multimorbidity—while minimizing the confounding effect of malnutrition through MNA screening.

Among the main limitations of this study are its retrospective, single-center design, which restricts the generalizability of the findings. Due to the cross-sectional nature of the study, a causal relationship between the HALP score and glycemic control could not be established. Frailty was not assessed using a validated multidimensional frailty scale; although the Barthel Index was used to evaluate functional dependence, this scale may not fully capture the complex and multidimensional nature of frailty. Additionally, the HALP score and HbA1c levels were measured only at baseline, precluding the evaluation of longitudinal changes. The relatively short three-month data collection period may have limited outcome variability and reduced the ability to capture longer-term changes in glycemic control. Furthermore, the AUC value of 0.645 indicates weak to modest discriminative ability, limiting the use of HALP as a standalone predictive marker for poor glycemic control. Another important limitation is the lack of data on several clinically relevant variables that may influence HbA1c levels, including diabetes duration, antidiabetic treatment regimens, insulin therapy, corticosteroid use, body mass index, and smoking status. The absence of these variables may have resulted in residual confounding and should be considered when interpreting the association between HALP and glycemic control. The lack of detailed data on medication adherence may have also influenced glycemic control. Finally, the absence of long-term clinical outcomes further limits the broader interpretation of the results.

## 5. Conclusions

In conclusion, the HALP score was independently associated with poor glycemic control in older adults with T2DM receiving home health care. This association remained significant in both the main multivariable model and the sensitivity analysis adjusted for clinically relevant variables, supporting the robustness of the findings. However, given its weak to modest discriminative ability, HALP should be interpreted alongside clinical assessment and routine metabolic parameters rather than as a standalone diagnostic or predictive criterion. Prospective multicenter studies with longitudinal follow-up are warranted to validate these findings and clarify the prognostic value of HALP in long-term glycemic control and diabetes-related outcomes.

## Figures and Tables

**Table 1:** Demographic and clinical characteristics of the study population.

Variable	*n* (%)/Median (Min–Max)
Age	83 (65–100)
Sex	
Male	105 (52.2)
Female	96 (47.8)
Hypertension	
Yes	188 (93.5)
No	13 (6.5)
Coronary artery disease	
Yes	58 (28.9)
No	143 (71.1)
Hyperlipidemia	
Yes	74 (36.8)
No	127 (63.2)
Asthma/COPD	
Yes	21 (10.4)
No	180 (89.6)
Presence of comorbidity	
Yes	196 (97.5)
No	5 (2.5)
Barthel Index	
Independent	12 (6.0)
Mildly dependent	8 (4.0)
Moderately dependent	44 (21.9)
Severely dependent	124 (61.7)
Totally dependent	13 (6.5)
HbA1c group	
Normal HbA1c (<8.0%)	150 (74.6)
Elevated HbA1c (≥8.0%)	51 (25.4)

Abbreviations: COPD, chronic obstructive pulmonary disease.

**Table 2:** Laboratory characteristics of the study population.

Parameter	Median (Min–Max)
HbA1c (%)	6.8 (4.7–12.0)
Hemoglobin (g/dL)	12.6 (6.9–16.6)
Albumin (g/L)	39 (23–53)
Lymphocyte (×10^9^/L)	1.89 (0.37–5.74)
Platelet (×10^9^/L)	244 (81–600)
Neutrophil (×10^9^/L)	4.62 (1.0–20.1)
Monocyte (×10^9^/L)	0.60 (0.2–4.6)
HALP score	37.4 (3.4–145.1)

Abbreviations: HALP, hemoglobin, albumin, lymphocyte, and platelet.

**Table 3:** Univariate and multivariable logistic regression analyses for elevated HbA1c levels.

Variable	Univariate OR (95% CI)	*p*	Multivariable OR (95% CI)	*p*
Age, per year	1.00 (0.95–1.04)	0.837	—	—
Sex	1.32 (0.70–2.49)	0.392	—	—
Barthel group	1.72 (0.83–3.57)	0.143	—	—
HALP (high vs. low)	0.27 (0.14–0.52)	<0.001	0.28 (0.14–0.54)	<0.001
Coronary artery disease	1.03 (0.52–2.08)	0.919	—	—
Asthma/COPD	0.66 (0.21–2.08)	0.484	—	—
Dementia	0.36 (0.12–1.06)	0.065	0.38 (0.12–1.18)	0.095

Abbreviations: CI, confidence interval; COPD, chronic obstructive pulmonary disease; HALP, hemoglobin, albumin, lymphocyte, and platelet; OR, odds ratio. Low HALP was defined as <29.24 and high HALP as ≥29.24 based on the ROC-derived Youden index cut-off. The odds ratios for HALP reflect the comparison of high HALP versus low HALP.

**Table 4:** Sensitivity analysis including clinically relevant variables for elevated HbA1c levels.

Variable	OR (95% CI)	*p*
Age, per year	0.99 (0.95–1.04)	0.793
Sex	1.03 (0.52–2.04)	0.934
HALP (high vs. low)	0.30 (0.15–0.59)	0.001
Barthel group	1.47 (0.68–3.17)	0.329
Dementia	0.38 (0.12–1.17)	0.091

Abbreviations: CI, confidence interval; HALP, hemoglobin, albumin, lymphocyte, and platelet; OR, odds ratio. This sensitivity model included clinically relevant variables regardless of their univariate statistical significance: age, sex, HALP group, Barthel group, and dementia. Low HALP was defined as <29.24 and high HALP as ≥29.24 based on the ROC-derived Youden index cut-off. The odds ratios for HALP represent the comparison of high HALP versus low HALP.

## Data Availability

The datasets are available from the corresponding author upon reasonable request.

## Abbreviations

The following abbreviations are used in this manuscript:

T2DM	Type 2 diabetes mellitus
HALP	Hemoglobin, albumin, lymphocyte, and platelet
NLR	Neutrophil-to-lymphocyte ratio
PLR	Platelet-to-lymphocyte ratio
SII	Systemic immune–inflammation index
COPD	Chronic obstructive pulmonary disease
